# Pragmatic Models for Detection of Hypertension Using Ballistocardiograph Signals and Machine Learning

**DOI:** 10.3390/bioengineering13010043

**Published:** 2025-12-30

**Authors:** Sunil Kumar Prabhakar, Dong-Ok Won

**Affiliations:** Department of Artificial Intelligence Convergence, Hallym University, Chuncheon 24252, Republic of Korea; sunilprabhakar22@gmail.com

**Keywords:** feature extraction, feature selection, classification, BCG, hypertension

## Abstract

To identify hypertension, Ballistocardiograph (BCG) signals can be primarily utilized. The BCG signal must be thoroughly understood and interpreted so that its application in the classification process could become clearer and more distinct. Various unhealthy habits such as excess consumption of alcohol and tobacco, accompanied by a lack of good diet and a sedentary lifestyle, lead to hypertension. Common symptoms of hypertension include chest pain, shortness of breath, blurred vision, mood swings, frequent urination, etc. In this work, two pragmatic models are proposed for the detection of hypertension using BCG signals and machine learning models. The first model uses K-means clustering, the maximum overlap discrete wavelet transform (MODWT) and the Empirical Wavelet Transform (EWT) techniques for feature extraction, followed by the Binary Tunicate Swarm Algorithm (BTSA) and Information Gain (IG) for feature selection, as well as two efficient hybrid classifiers such as the Hybrid AdaBoost–-Maximum Uncertainty Linear Discriminant Analysis (MULDA) classifier and the Hybrid AdaBoost–Random Forest (RF) classifier for the classification of BCG signals. The second model uses Principal Component Analysis (PCA), Kernel Principal Component Analysis (KPCA) and the Random Feature Mapping (RFM) technique for feature extraction, followed by IG and the Aquila Optimization Algorithm (AOA) for feature selection, as well as two versatile hybrid classifiers such as the Hybrid AutoRegressive Integrated Moving Average (ARIMA)–AdaBoost classifier and the Time-weighted Hybrid AdaBoost–Support Vector Machine (TW-HASVM) classifier for the classification of BCG signals. The proposed methodology was tested on a publicly available BCG dataset, and the best results were obtained when the KPCA feature extraction technique was used with the AOA feature selection technique and classified using the Hybrid ARIMA–AdaBoost classifier, reporting a good classification accuracy of 96.89%.

## 1. Introduction

The BCG is an estimate of ballistic potencies produced and initiated by the heart [1]. With every heartbeat, when the blood is ejected suddenly into the great vessels, the motions of the human body have a repetitive format and are represented graphically with the help of the BCG [2]. The utilization of the BCG lies in the use of a ballistographic scale, where the recoil of the body of the patient can be measured and analyzed. The heart rate of a person can be exhibited very well with the help of a BCG scale [3]. The pumping action of the heart and other body movements can be apprehended well with the help of non-invasive sensors, and some mechanical forces are generated and recorded, known as BCG signals [4]. With every heartbeat, the ejection of the blood is performed into the aorta when the recoil of the body is generated, and this is represented as an actual form of BCG. The BCG signals help to capture the heartbeat forces, vital sign monitoring and other body movements. Non-invasive sensors are utilized to record BCG signals. Some of the sensor types used to analyze the BCG signal include fiber optic sensors, strain gauge, piezoelectric, gyroscope, pneumatic, hydraulic, polyvinylidene and electromechanical film sensors, etc. [5]. The BCG signals are subject to data processing such as filtering and amplification before being processed for full-fledged analysis. Some of the wonderful applications of BCG include non-obtrusive monitoring, sleep tracking, infant monitoring and other clinical applications, including stroke volume estimation and blood pressure estimation [6]. As with other biomedical signals, BCG signals also are subject to a lot of problems, such as signal noise, standardization issues and data interpretation problems.

The heartbeat-prompted motions of the human body, which are continuously repeated, can be graphically represented with the help of this non-invasive technique. During the systole and diastole periods of heartbeat, the blood accelerates rapidly, leading to these motions being repeated incessantly. The overall execution of the circulatory system can be assessed well with the help of BCG. The mass movements of the cardiac cycle are measured and analyzed by this technique. When the systolic and diastolic phases occur, a lot of components can become shifted due to the changes happening in the body movements, cardiac activity and respiratory nodes [7]. Because of these movements, the BCG waveform is generated, as there would be a significant change in the distribution of blood during this cardiac cycle. Earlier BCG did not have a proper and standardized measurement protocol; moreover, the origin of this waveform was not understood well [8]. For clinical diagnosis of cardiovascular disorders such as myocardial infarction and coronary artery diseases, a high level of reliability and specificity is required, which was not reached by BCG at that time. Moreover, more advanced techniques such as echocardiography, photoplethysmography (PPG) and ultrasound proved to be a success, as they provided a comparatively high level of reliability and sensitivity to medical researchers who work in this domain [9]. Currently, due to the advent of both software and hardware technologies, a lot of interest has been received by BCG. BCG sensors can be embedded in medical environments easily, even without the involvement of medical professionals or the general public. Nowadays, the stress associated with medical checkup for patients has been greatly reduced. With the help of multiple technological leads, the concept of BCG has been received well in the past two decades [10]. Therefore, the concept of BCG is highly useful for tracking sleep-related disorders, managing respiratory disorders and monitoring cardiac functions. Moreover, the easy availability and accessibility of BCG allow the public to employ it in their homes for tracking the daily activities of the patient.

Genetics and other environmental factors play a vital role in hypertension [11]. Reducing stress and improving one’s lifestyle by following a good diet and exercise pattern can improve the symptoms of hypertension. When the heart pumps, the BCG signals can be generated, and there is a good correlation in detecting hypertension or any cardiovascular disorder associated with it [12]. The vital information about the blood volume pumped by the heart per 60 s can be obtained with the help of BCG signals. The elevated blood pressure can be described well by the BCG signals, and solid data can be provided about the blood vessel stiffness too [13]. A lot of important challenges are faced by the healthcare systems these days. To improve healthcare quality, a lot of invasive and non-invasive techniques have been developed by researchers, largely considering the effectiveness and cost factors, and in this aspect, BCG plays quite an important role in improving the health quality of the patients to a great extent. The morphology of the BCG signal varies greatly within the subjects, and so BCG signal processing is quite a challenging task [14].

An in-depth study of BCG covering its theory and developments was discussed in detail by Pinheiro et al. [15]. For the analysis of a medical cardiovascular physiology system, a BCG system was developed by Antonio [16], and a comparative study was conducted by Sadek and Biswas [17] based on the non-intrusive heart rate measurement utilizing the BCG signals. The BCG peak detection techniques were discussed in detail by Suliman et al. [18], and various clustering techniques utilized to detect the heartbeat in the BCG signals were elaborated by Paalasmaa and Ranta [19]. In a moving wheelchair equipped with embedded sensors, the acquisition of BCG was studied by Pinheiro et al. [20], and a maximum dispersion technique was used for the detection of a simple real-time heartbeat in BCG signals by Choe et al. [21]. A versatile BCG acquisition system was developed for home monitoring purposes by Inan et al. [22], and the motion artifacts were detected automatically in the BCG based on a modified bathroom scale by Wiard et al. [23]. The heart rate estimation based on an adaptive beat-to-beat basis for the BCG signals was developed by Bruser et al. [24], and a novel technique for BCG measurements utilizing fiber Bragg grating-based sensors was developed by Lukasz et al. [25]. The Phonocardiogram (PCG), Electrocardiogram (ECG) and BCG signals were thoroughly compared for the application of heart rate monitoring of the human body by Nedoma et al. [26].

A lot of the recent important works for the detection of hypertension using BCG signals are discussed as follows. Using ensemble empirical mode decomposition (EMD), the detection of hypertension was performed using BCG signals, and an accuracy of 92.3% was reported [27]. The mining class association, along with many features was used to identify the hypertension by Liu et al., achieving an accuracy of 85.20% [28]. By means of integrating association rule mining and classification rules, hypertension could be identified by Liu et al. with an accuracy of 84.40% [29]. A deep CNN architecture with two stages was used for the classification of low-risk versus high-risk hypertension using ECG signals, and a higher accuracy of 99.68% was obtained [30]. The EMD with wavelet decomposition and ensemble gentle-boost classifier was used by Rajput et al., achieving an accuracy of 89% [31]. A continuous wavelet transform with deep neural networks (DNN) was used by Rajput et al., achieving an accuracy of 86.14% [32]. A Gabor transform-based deep convolutional neural network (CNN) was used for the automated detection of hypertension by Gupta et al., achieving an accuracy of 97.65% [33]. Depending on the time–frequency domain and deep learning features, pulmonary hypertension was detected using a PCG dataset, achieving a classification accuracy of 88.61% [34]. The power-normalized cepstral coefficients, along with CNN, were used for the analysis of congenital heart disease-related pulmonary arterial hypertension using heart sound signals, achieving an accuracy of 88.61% [35]. A deep CNN technique was used for hypertension on pregnancy disorders achieving an accuracy of 93.10% [36]. A novel odd–even pattern was utilized for the detection of hypertension using BCG signals, achieving an accuracy of 97.78% [37]. A CNN was used with fine-tuned spectrogram images and ConvMixer model, achieving an accuracy of 97.69% [38]. An integrated tunable Q-factor wavelet transform along with the concept of multi-verse optimization was proposed by Gupta et al., achieving a classification accuracy of 92.21% [39]. In this work two interesting pragmatic models were proposed for the analysis and classification of hypertension from BCG signals. The organization of this work is as follows:

Once the basic pre-processing of the BCG signals is performed by means of implementing a simple Independent Component Analysis (ICA), it is then processed with the help of two proposed pragmatic models.

The first model uses the idea of K-means clustering, MODWT and EWT for feature extraction followed by the usage of BTSA and IG for feature selection and two hybrid classifiers such as Hybrid AdaBoost–MULDA classifier and Hybrid AdaBoost–Random Forest classifier.

The second model uses the PCA, KPCA and the RFM technique for feature extraction, followed by the usage of AOA and IG for feature selection and two hybrid classifiers, such as the Hybrid ARIMA–AdaBoost classifier and the TW-HASVM classifier.

The workflow implemented in this paper is the first of its kind, as no work in the past has adopted such a workflow employing such a hybrid combination to test its versatility on BCG signal analysis.

**Figure 1:**
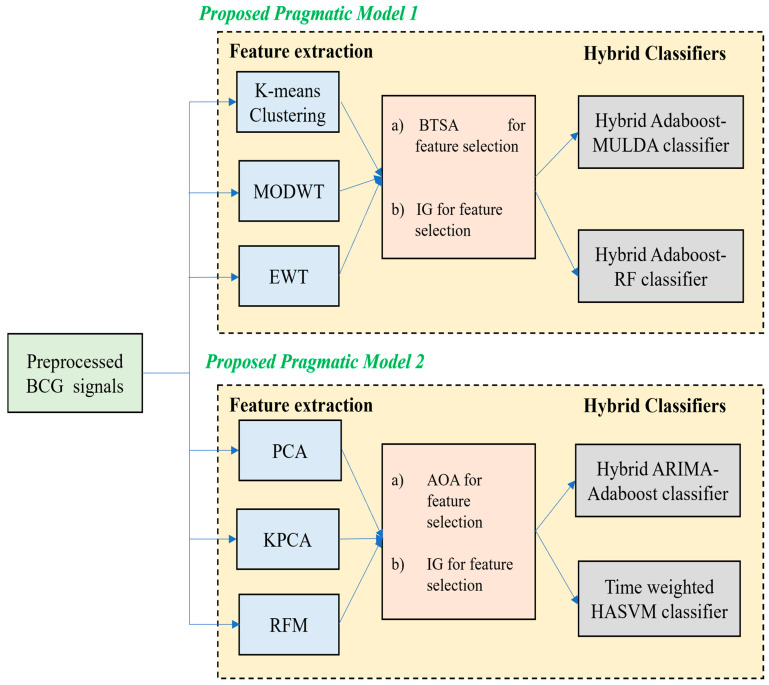
Illustration of the two proposed pragmatic models.

The overall pictorial representation of the work is illustrated in Figure 1 below.

## 2. Proposed Pragmatic Model 1

The first model uses the idea of K-means clustering, MODWT and EWT for feature extraction, followed by the usage of BTSA and IG for feature selection and two hybrid classifiers, such as the Hybrid AdaBoost–MULDA classifier and the Hybrid AdaBoost–Random Forest classifier.

### 2.1. Feature Extraction Using K-Means Clustering, MODWT and EWT

#### 2.1.1. K-Means Clustering

For the categorization of unlabeled data, K-means clustering is used so that the cluster centers can be located easily [40]. In an iterative manner, the cluster centers are changed by this K-means algorithm, and so the overall variation can be mitigated amongst these clusters. The distance between the center of a cluster and its respective data can be reduced drastically by assigning observations to the K-clusters. When there is a change in the group center, the redistribution of the data points happens and then the computation of group center can be performed easily. The grouping of the entire data can be carried out into specific numbers of K categories. The basic procedure for utilizing the K-means algorithm is as follows:

In a random manner, the creation of the K data points is performed so that the primary cluster centers can be created.

The computation of the distance of every data point from the cluster centers is performed.

The assignment of the data point to any of the neighboring clusters is performed depending on a proximity basis.

Assume a set of q-dimensional vectors with the notation $\left\{P_{i}\right\}=\left\{p_{i1},p_{i2},\ldots ,p_{iq}\right\}$. The square of the Euclidean distance usually assesses the dissimilarity measure and is expressed as follows:

(1) $$d\left(p_{i},p_{j}^{\prime }\right)={\sum_{j=1}^{q}\left(p_{ij}-p_{ij}\right)}^{2}={\left\|p_{i}-p_{j}^{\prime }\right\|}^{2}$$

Within a particular cluster, the data is assigned, and the establishment of a new cluster center is determined.

For every cluster, the process is repeated until the progress is quite satisfactory.

#### 2.1.2. Maximal Overlap Discrete Wavelet Transforms (MODWT)

With the help of three unique stages, the MODWT can be explained as Discrete Wavelet Transform (DWT), Discrete Wavelet Packet Transform (DWPT) and, finally, MODWT [41]. An incoming signal can be evaluated easily with this transformation. Initially, DWT helps to decompose the input signal so that the estimates can be obtained correctly. An incoming signal is split by DWT into scaling coefficients and wavelet coefficients. Secondly, when a gradual refinement of DWT happens, it is projected as DWPT [42]. The modification of the DWT pyramid technique is performed as if it was an orthonormal transformation. At a specific level, both the wavelets and scaling coefficients are decomposed by DWPT, and so the prediction of parameters becomes quite easy. Some of the decomposition coefficients can be easily lost with the help of DWT and DWPT methods, as they are highly time-variant transforms. A time-invariant transformation is therefore created so that the wavelet values are aligned correctly with the input parameters and it is performed without the aid of down-sampling method. In MODWT, the input signal is split with the help of high- and low-pass filters, and the coefficients are obtained, which would have a good pass-band period during the decomposition stage [41]. The input signal length becomes similar to the coefficient’s length when the down-sampling is eliminated. Every produced decomposed constituent finally has a corresponding time series, which is strongly linked to the others.

#### 2.1.3. Empirical Wavelet Transform (EWT)

In different areas of data sciences, one of the most powerful signal processing tools implemented is EWT [43]. The implementation of fixed basis functions is restricted mostly in wavelet transformations. The signal decomposition can be performed by EMD to a certain extent, where the oscillatory components could be utilized to highlight the properties of the input signal. When using the EMD, the derivation of the basis function is quite easy from the input signal. Though EMD has proved its mettle in various applications, it still has some drawbacks due to the lack of a strong mathematical foundation, so EWT came into existence. EWT uses the properties of both EMD and WT. A time-series signal is transformed by EWT decomposition into a collection of time series, which exhibits a stable variance when compared with the original signal, so the ultimate result would be more accurate and precise. Fourier segments can be assessed easily with the help of EWT, and so several wavelet filters can be constructed easily so that different modes can be extracted from the time-series signal. When the specification of Fourier segments is given, the application of EWT occurs. The local maxima of the spectrum are located and designed by this empirical rule. Finally, the Fourier segments boundaries are used and projected as the midpoint of the adjacent local maxima.

### 2.2. Feature Selection Using Binary Tunicate Swarm Algorithm (BTSA)

One of the famous swarm-based optimization techniques is TSA, where the search mechanism of food source of tunicates is mimicked in the deep ocean [44]. With the help of a jet propulsion scheme, all the tunicates progress towards food sources, and an extreme level of intelligence is exhibited. To solve an optimization issue, the above behavior of tunicates is modelled mathematically. The tunicates’ position is generally assigned to a binary vector in the case of BTSA. The tunicates exhibit a wonderful swarm behavior, which enables them to move towards the best tunicate. A good proximity is maintained among the tunicates and, at the same time, collision is avoided among them in a careful manner. The mathematical modelling of the tunicate behavior is performed as follows.

When the tunicates search for food, they try to avoid a lot of collision, and certain parameters are utilized to control this behavior and are represented as follows:

(2) $$\vec{T}=\frac{\vec{G}}{\vec{S}}$$

where the gravitational force is expressed by $\vec{G}$, and the social force is expressed by $\vec{S}$, which largely helps to affect the tunicate movement. With the aid of water-flow advection $\left(\vec{W}\right)$, the control of the gravitational force is performed as follows:

(3) $$\vec{G}=k_{1}+k_{2}-\vec{W}$$

(4) $$\vec{W}=2k_{3}$$

where $k_{1},k_{2},k_{3}$ are represented as random parameters represented in the range of [0, 1]. The parameter $\vec{S}$ is expressed as follows:

(5) $$\vec{S}=w_{i}+k_{3}\left(w_{m}-w_{i}\right)$$

where the $w_{i}$ expresses minimum speed, and $w_{m}$ expresses maximum speed so that a large number of social interactions are made. The tunicate helps to find the best position in the process. To avoid collision, a distance is maintained among the remaining tunicates, and the distance is expressed as follows:

(6) $${\vec{D}}_{h}=\left|\vec{B_{q}}-r_{1}.\vec{B_{h}}(i)\right|$$

where $D_{h}$ represents the distance of current location of the $h^{th}$ tunicate $\left(\vec{B_{h}}(i)\right)$, and $r_{1}$ represents a random number in the range of [0, 1]. Around the best tunicate, the remaining tunicates remain, and they trace the optimal position of the food source. This mechanism is expressed as follows:

(7) $${\vec{B}}_{h}(i)=\left\{\begin{array}{c} \vec{B_{q}}+\vec{T}\cdot \vec{D_{h}},rand\geq 0.5\\ \vec{B_{q}}-\vec{T}\cdot \vec{D_{h}},rand\leq 0.5 \end{array}\right.$$

The updating of the $h^{th}$ tunicates position is expressed as follows:

(8) $${\vec{B}}_{h}(i+1)=\frac{\vec{B_{h}}(i)+\vec{B_{q}}}{2+k_{1}}$$

To match the feature selection issues, the conversion of these positions into a binary value is quite mandatory. By means of implementing various transfer functions, such as V-shaped and S-shaped, this conversion occurs. Generally, it is observed that the efficiency is much higher in the V-shaped transfer function when compared to the S-shaped transfer function. With the help of the V-shaped transfer function, the design of BTSA is performed. The positions are updated in the subsequent iteration from 0 to 1 with the help of the transfer function. The updating of the tunicate position in the BTSA is expressed as follows:

(9) $$T.F(t)=\left|\frac{2}{\pi }\arctan\left(\frac{\pi }{2}t\right)\right|$$

where T.F represents a transfer function.

(10) $${\vec{B}}_{h}(i+1)=\left\{\begin{array}{c} \vec{B_{h}}{\left(i\right)}^{\prime },rand\left(\;\right)<T.F\left(\vec{B_{h}}(i+1)\right)\\ \vec{B_{h}}(i),rand\left(\;\right)\geq T.F\left(\vec{B_{h}}(i+1)\right) \end{array}\right.$$

where ${\vec{B}}_{h}{\left(i\right)}^{\prime }$ indicates the complement of ${\vec{B}}_{h}(i)$.

The fitness function here depends on Feature Selection Ratio (FSR) and classification error, and is designed as follows:

(11) $$Fitness=k_{1}*error+k_{2}*FSR$$

The original classification error is denoted by error. The weighting factors are indicated by $k_{1}$ and $k_{2}$, falling in the range of [0, 1]. To have a lower classification burden, a lower FSR is generally required.

### 2.3. Classification Using Two Hybrid Models

Two hybrid classifiers are proposed, such as the Hybrid AdaBoost–MULDA and the Hybrid AdaBoost–RF classifiers.

#### 2.3.1. Hybrid AdaBoost–MULDA Classifier

(A) MULDA:

For the non-dominant values, the estimation errors are higher and moreover the impact of the principal components largely affects the classification accuracy. To address this issue, MULDA is usually used in literature [45]. In MULDA, the smaller eigen values can be enlarged well so that the larger eigen values do not change. The instabilities can be reduced greatly by MULDA so that the identity matrix can be stabilized well thereby managing the within-class covariance matrix in an efficient manner. For stabilizing the identity matrix, choosing the maximum entropy technique is highly essential. Assuming a data comprising of n samples and is split into ‘*c*’ number of classes. Assume $P=\left\{p_{1},p_{2},\ldots ,p_{n}\right\}\in {ℜ}^{d}$ indicate the sample mean, $\bar{p}$ specifies the sample mean in a class. The MULDA procedure is implemented as follows:

Compute the within class matrix and between class matrix $M_{w}$ and $M_{b}$.

Calculate $M_{q}=M_{w}/\left(n-c\right)$.

Compute the eigenvector matrix φ and eigenvalue diagonal matrix Λ.

Assess the eigenvalues λ of $M_{q}$.

Assess the average eigenvalues $\bar{\lambda }$.

New eigenvalues matrix $\Lambda^{*}$ is generated by replacing λ with $\bar{\lambda }$.

Compute the within-class scatter matrix as: $M_{w}^{*}=M_{q}^{*}(n-c)=\left(\varphi \wedge^{*}\varphi^{T}\right)\left(n-c\right)$.

The discriminant transformation vector is obtained ultimately by $M_{w}^{*-1}M_{b}$.

For the training samples, $\left(c-1\right)$ discriminant transformation vectors are calculated. Then the projection of test samples on the training samples is performed in the subsequent stage. Using a KNN algorithm, the performance of classification is assessed and finally the effectiveness of this validation is evaluated to serve the classification accuracy.

(B) Hybrid AdaBoost–MULDA classification:

AdaBoost helps to transform many weak classifiers into one strong classifier [46]. MULDA is hybrid with this concept so that better classification accuracy could be obtained. A lot of weight adjustments are performed iteratively and misclassified samples are given more importance in this hybrid model. The new weak classifiers are trained iteratively and so the misclassified samples are prioritized greatly, so that a best predictive classifier can be obtained. For diverse classification tasks, AdaBoost classifier is used as it could very well handle high dimensional data in a versatile manner. For these reasons, MULDA is hybrid with AdaBoost so that it could develop as a strong classification model. Across all the samples, the weight distribution is split equally to all the combinations. The classification error is then computed so that the weights can be adjusted normally. Increased weightage is given to the misclassified samples and less weightage is given to the correctly classified samples. So, the primary focus is on error-prone samples by means of iteratively adjusting the weights leading to multiple MULDA classifiers. Each sample must be concentrated keenly so that the effectiveness of every classifier is projected clearly as the performance output measure. In this Hybrid AdaBoost-MULDA classification model, the effect of every MULDA classifier is assessed by the classification accuracy. This model ensures and strives to bring out the best outcome in terms of classification accuracy.

#### 2.3.2. Hybrid AdaBoost–RF Classifier

An ensemble machine learning technique can be modelled with the help of AdaBoost classifier and RF technique [47]. The reassignment of every weight of the value can be applied to each instance which has a higher weight. Various weak classifiers can be merged so that a robust classifier can be constructed easily. There is a less necessity for tweaking parameters and it is easy to implement as it is not prone to over fitting. The simple pseudocode for the AdaBoost algorithm is expressed in Algorithm 1 as follows:

**Algorithm 1:** AdaBoost ClassificationAssume: $\left(a_{i1},b_{i1}\right)\ldots \ldots \left(a_{iz},b_{iz}\right),a_{ii}\in A_{i},b_{ii}\in \left\{-1,1\right\}$ Start Dii(ii)=1zi For $t_{i}=1,\ldots ,T_{i}$ Weak classifier training phase using the distribution $D_{i}$ Obtain weak hypothesis $h_{ii}:A_{i}\to \left\{-1,1\right\}$ Compute error $\Sigma_{ii:h_{it}\left(a_{ii}\right)\neq b_{ii}}D_{it}\left(a_{ii}\right)$ Choose $\alpha_{it}=\frac{1}{2}\log\left(1-\frac{\varepsilon_{ti}}{\varepsilon_{ti}}\right)$ Updating all the parameters $D_{it+1}(ii)=\frac{D_{it}(ii)}{N_{t}}=\left\{\frac{e^{-\alpha t}\;if\;instance\;ii\;is\;classified\;correctly}{e^{\alpha t}\;if\;instance\;ii\;is\;not\;classified\;correctly}\right\}$ where $N_{t}$ denotes the normalization factors and it is presumed as $\sum_{ii=1}^{m_{i}}D_{it+1}=1$ Output the hypothesis $H_{i}(a_{i})=sign\left(\sum_{t_{i}-1}^{T}\alpha_{it}h_{it}\left(a_{ii}\right)\right)$

(A) RF Implementation:

One of the most used ML algorithms is RF. A single outcome can be obtained by the hybridization of different decision trees in this concept of RF. Both classification issues and regression problems can be tackled well with the help of RF. The performance of any model can be enhanced by merging different classifiers so that more complex problems can be solved efficiently. The prediction of the correct output cannot be performed by decision trees alone, as generally a high number of trees are required to build the model, resulting in high computational complexity. The pseudocode for RF is expressed in Algorithm 2.

**Algorithm 2:** RF ClassificationInput: Training samples Weight initialization by random values; $W_{ii}=Value_{random}$ Compute the sum of inputs $\left(A_{i1},A_{i2},A_{i3},\ldots ,A_{in}\right)$: $S_{hi}:=\sum_{i=1}^{n}A_{ii}.W_{ii}+c_{i}$ Multiply with weights $\left(W_{i1},W_{i2},W_{i3},\ldots ,W_{in}\right)$ for the hidden layer node Project the hidden output for hidden layer node The input for the output layer nodes is expressed as $S_{out}:=\sum_{i=1}^{n}A_{ii}.W_{ii}+c_{i}$ Project the output at output nodes The difference between the predicted output and actual output is computed so that the error rate is obtained The error rate is detected from hidden layer to output layer nodes The updating of the network weights is performed The final output is the difference between the predicted output and actual output

(B) Hybrid AdaBoost–RF implementation:

The weak learners can be converted to strong learners easily with the help of AdaBoost and RF algorithms. To enhance accuracy and to improve the stability, RF is hybridized with AdaBoost so that the high weight instances are easily chosen. The training of RF algorithm is easier, but once the training process is complete, it becomes slow; therefore, to enhance speed, the AdaBoost algorithm is hybridized with RF. By hybridizing AdaBoost and RF, training time can be reduced easily so that better accuracy can be obtained. The RF estimation is widely used, leading to an accurate classification by largely minimizing the losses. Both the weak points and strong points of the hybrid algorithm are examined so that the obtained error rate is much lower. The algorithm for the Hybrid AdaBoost–RF algorithm is expressed in Algorithm 3 as follows:

**Algorithm 3:** Hybrid AdaBoost–RF classificationGiven $\left(a_{i1},b_{i1}\right)\ldots \left(a_{iz},b_{iz}\right),a_{ii}\in A_{i},b_{ii}\in B_{i}=\left\{-1,1\right\}$ Assign Dii(ii)=1zi For $t_{i}=1,\ldots ,T_{i}$ Train the weak classifiers by using distribution $D_{i}$ Weak hypothesis is fetched $h_{ii}:A_{i}\to \left\{-1,1\right\}$ Compute error $\sum_{ii:h_{it}}\left(a_{ii}\right)\neq b_{ii}D_{it}\left(a_{ii}\right)$ Choose $\alpha_{it}=\frac{1}{2}\log\left(1-\frac{\varepsilon_{ti}}{\varepsilon_{ti}}\right)$ Increment the hypothesis if required Compute the sum of the inputs as $S_{h_{i}}:=\sum_{i=1}^{n}A_{ii}.W_{ii}+c_{i}$ Multiply $\left(A_{i1},A_{i2},A_{i3},\ldots ,A_{in}\right)$ with $\left(W_{i1},W_{i2},W_{i3},\ldots ,W_{in}\right)$ for the hidden layer nodes Compute the hidden output for hidden layer node Input for the output layer node is given as $S_{out}:=\sum_{i=1}^{n}A_{ii}.W_{ii}+c_{i}$ Compute the distribution as $D_{it+1}(ii)=\frac{S_{out}(ii)}{N_{t}}=\left\{\frac{e^{-\alpha t}\;if\;instance\;ii\;is\;classified\;accurately}{e^{\alpha t}\;if\;instance\;ii\;is\;not\;classified\;accurately}\right\}$ where the normalization factor $N_{t}$ is expressed as $\sum_{ii=1}^{z_{i}}D_{it+1}=1$ Output the final hypothesis $H_{i}(a_{i})=sign\left(\sum_{t_{i-1}}^{T}\alpha_{it}h_{it}\left(a_{ii}\right)\right)$

## 3. Proposed Pragmatic Model 2

The second model uses the PCA, KPCA and RFM techniques for feature extraction, followed by the usage of AOA and IG for feature selection and two hybrid classifiers such as the Hybrid ARIMA–AdaBoost classifier and the TW-HASVM classifier.

### 3.1. Feature Extraction Using PCA, KPCA and RFM Techniques

#### 3.1.1. PCA and KPCA

To handle nonlinear relationships efficiently, the traditional PCA can be extended with the aid of kernels. It is widely used in multiple applications such as remote sensing, Natural Language Processing (NLP), image processing etc. As raw data has a lot of redundant features, KPCA is utilized to largely remove them [48]. Initially, the ideal kernel function must be selected. Various kernels, such as Gaussian, Polynomial, Radial Basis, sigmoid, etc., are available, and in this work, a Gaussian kernel is chosen. Then, the kernel matrix is computed so that the pairwise similarity is specified correctly between the data points with the help of kernel function. The kernel matrix is a simple n×n symmetric matrix. If the total number of data points is represented as n, the kernel function is represented as follows:

(12) $$K\left(p_{i},p_{j}\right)=\left(p_{i},p_{j}\right)$$

Then, eigendecomposition is implemented so that the eigenvectors and eigenvalues are obtained. In the high-dimensional space, the principal components are the simple eigenvectors. Eigenvalues help to assess the total variance of every component. The eigenvector $E_{v}$ is indicated as follows:

(13) $$E_{v}=\sum_{j=1}^{n}\beta_{j}\mu \left(p_{j}\right)$$

where the training samples are represented by $p_{j}$, and the eigenvectors are represented by $E_{v}$. The K eigenvectors related to the K eigenvalues are chosen. The vital principal components are specified by the eigenvectors so that a majority of variance is captured in the entire data. For extracting the entire features, the chosen principal components are the original projection of the entire data. Thus, the essential structure of the data is preserved and, at the same time, the overall dimension of the data is greatly mitigated. The derivation of the high dimensional feature space is expressed as follows:

(14) $$\left({e_{v}}^{l}.\mu \left(p_{i}\right)\right)={\sum_{j=1}^{n}\left(\beta_{j}\right)}^{l}K\left(p_{j},p_{i}\right)$$

where the principal components are expressed by $\beta_{j}$; the feature vectors are represented as ${e_{v}}^{l}$, and the mapping coefficient is denoted by μ. The inherent patterns are captured, and the extracted features are effectively obtained by implementing the KPCA model. From these extracted features, the most suitable features can be selected and fed for classification to obtain a high classification accuracy.

#### 3.1.2. Random Feature Mapping Technique

A dataset of various dimensions must be processed, and so RFM is utilized in this work [49]. On the input samples, the linear transformation is incorporated so that feature nodes are formed. The stacking of feature nodes is performed so that a feature mapping layer is formed which aids in feature extraction. A random feature mapping technique usually has an input layer and a feature mapping layer. The input data features equal the total number of neurons in the input layer. If ‘*K*’ number of features are present in the input data, the vector $g_{i}=\left(g_{i1},g_{i2},\ldots ,g_{ik}\right)$ is utilized to specify the $i^{th}$ input data. The matrix $G={\left(g_{1},g_{2},\ldots ,g_{i},\ldots ,g_{n}\right)}^{T}$ helps to identify the n K-dimensional input vectors from the input data. The batch processing of the input data G is performed and then for transformation purposes, it is progressed to the feature mapping context zone. The normalization of the original input data is performed so that it has a variety of properties. Assuming ‘*q*’ group nodes are present in the feature nodes of the feature mapping, they are larger and represented as $Z_{i}\;(i=1,2,\ldots ,q)$, which has a collection of ‘*n*’ numbers of neurons. Once the feature mapping of $Z_{i}$ is performed successfully, G is expressed as follows:

(15) $$Z_{i}=\varphi_{i}\left(GW_{e}+\beta_{i}\right)$$

where the activation function is activated by $\varphi_{i}$; in this work, the ReLU function is used. The weight matrix is indicated by $W_{i}$, and the bias matrix is specified by $\beta_{i}$. A random generation of the initial values can be performed successfully in the weight matrix and the bias matrix, and it is represented as follows:

(16) $$W_{i}\in {ℜ}^{M\times n}$$

(17) $$\beta_{i}\in {ℜ}^{Q\times n}$$

The random features must be fine-tuned slightly into a new set of sparse features, and this is enabled by sparse autoencoders. For the generated new feature nodes, the linear correlation is reduced drastically. From the input data, the sparse features must be extracted, and the lasso technique is implemented so that the optimization problem can be solved easily. To fine-tune $W_{i}$ and $\beta_{i}$, a sparse matrix is assigned, and the objective function for optimization purposes is assigned as follows:

(18) $$\underset{W_{i}}{\arg\min}:{\left\|ZW_{i}-G\right\|}_{2}^{2}+\gamma {\left\|W_{i}\right\|}_{1}$$

A lot of restrictions can be imposed on the weight matrix by γ. Thus, the important features are explored by sparse autoencoders, and the randomness can be easily overcome. For the data, the distribution specifications are fully reflected and identified by the generated feature subsets so that the generalization aspects of the model are largely improved.

### 3.2. Feature Selection Using Information Gain (IG)

A famous technique to evaluate the significance of features with respect to target variables is IG [50]. It has multiple advantages, including independence, robustness, speed and simplicity. The uncertainty is tried and removed by the information entropy and when the categorization occurs, each category is indicated by some random variable. Assuming the category to be $P=\left\{p_{1},p_{2},\ldots ,p_{i}\right\}$, then for the classification task, the information entropy is mathematically expressed as follows:

(19) $$H(P)=\sum_{i=1}^{n}\mathrm{Pr}\left(p_{i}\right)h_{2}\frac{1}{\mathrm{Pr}\left(p_{i}\right)}=-\sum_{i=1}^{n}\mathrm{Pr}\left(p_{i}\right)h_{2}\left(\mathrm{Pr}\left(p_{i}\right)\right)$$

where the number of classes is represented by n, and the probability is represented by Pr. For each feature, the IG needs to be computed. Assuming the feature as F and its respective values as $\left(f_{1},f_{2},\ldots ,f_{k}\right)$, for a class P, the conditional entropy is expressed as follows:

(20) $$H\left(\left.P\right|F\right)=-\sum_{k=1}^{l}\mathrm{Pr}\left(f_{k}\right)\sum_{i=1}^{n}\mathrm{Pr}\left(\left.p_{i}\right|f_{k}\right)\log_{2}\left(\mathrm{Pr}\left(\left.p_{i}\right|f_{k}\right)\right)$$

where the average uncertainty of class p is expressed by $H\left(\left.P\right|F\right)$. When a feature F is fixed, the conditional probability of class p is indicated by $\mathrm{Pr}\left(\left.p_{i}\right|f_{i}\right)$. For a feature F, the IG is indicated as the difference between a priori entropy $H\left(P\right)$ and conditional entropy $H\left(\left.P\right|F\right)$ and is expressed as follows:

(21) $$IG(F)=H(P)-H\left(\left.P\right|F\right)$$

### 3.3. Feature Selection Using AOA

For certain parameters, optimal values can be easily determined by the process of optimization. The best solutions cannot be assessed exactly with the help of mathematical programming methods, as the problem might display unrestricted specifications sometimes. Therefore, to trace the unknown search spaces and to seek the local optima, optimization techniques are usually proposed, and over the past few decades, meta-heuristic algorithms have gained considerable attention. Exploration and exploitation are the two important stages that all the meta-heuristic algorithms come across and a good balance should be framed between these two aspects always. The hunting behavior of the Aquila bird is mimicked by this nature-inspired metaheuristic algorithm [51]. Aquila has excellent hunting abilities and it targets animals such as hares, squirrels, rats, etc. These birds have very large territories, and they build their nest in very high places so that they can raise their young ones peacefully. When present in an isolated environment, a male Aquila can become more aggressive and can attack even an adult deer successfully. All kinds of rodents are generally a staple part of their diet. Four specific hunting techniques are employed by Aquila and depending on the situation, they can adopt and change their hunting methodology. The initial technique involves flying high in sky with a faster movement to attack the flying birds. The second technique involves hunting the target by flying at a low-level descent or altitude. The third technique involves the targeted attack of the prey, which are relatively slow-moving such as tortoises and snails. The final techniques involve the grabbing method of attack, where they usually pull the target out from hiding and then kill it. Based on these techniques, the AOA was developed. As Aquila is a relatively skillful hunter, the modelling of its different strategies can be expressed as follows:

***Technique 1:*** This phase could be called as expanded exploration phase. At the start, the information about its target can be identified by the Aquila, and then an exhaustive search strategy is used to accurately locate the position and is represented as follows:

(22) $$Y_{i}(t+1)=Y_{best}(t)\times \left(1-\frac{t}{T}\right)+Y_{p}(t)-Y_{best}(t)*r$$

The individual positions i is expressed by $Y_{i}(t+1)$ in the next (t+1) iteration. In the current iteration, the best optimal location is identified by $Y_{best}(t)$. In the current iteration, for every individual, the average position is projected by $Y_{p}(t)$ and is expressed as follows:

(23) $$Y_{p}(t)=\frac{1}{N}\sum_{i=1}^{N}Y_{i}(t)$$

At an iteration t, the position of the $i^{th}$ individual is expressed by $Y_{i}(t)$. The entire swarm population size is expressed by N. Here, a random number is assigned, which is a Gaussian distribution and is within the range [0, 1]. The highest iteration number is depicted by T.

***Technique 2:*** This phase could be called a focused exploration phase. When this phase occurs, the prey is located by aquila, and so it circles around the target and prepares to land to attack the prey, and it can be mathematically expressed as follows:

(24) $$Y_{i}(t+1)=Y_{best}(t)\times Levy(D)+Y_{R}(t)+\left(z-y\right)*r$$

Here, r is expressed as the random number, the dimensionality is indicated by D and Levy(D) helps to compute the Levy flights and is expressed as follows:

(25) $$Levy(D)=c\times \frac{\mu \times \sigma }{{\left|v\right|}^{\frac{1}{\beta }}}$$

where a constant parameter is represented by c and has a value of 0.1 in our experiment. The random numbers are again initiated to μ and v, and are assigned within the range of [0, 1]. The computation of σ is performed as follows:

(26) $$\sigma =\frac{\gamma \left(1+\alpha \right)\times \sin\left(\frac{\pi \alpha }{2}\right)}{\gamma \left(1+\alpha \right)\times \alpha \times 2^{\frac{\alpha -1}{2}}}$$

α is, again, a constant parameter and is set as 0.5 in our experiment, while the gamma factor is represented by γ.

$Y_{R}(t)$ indicates a randomly chosen candidate present at the current iteration. With the help of variables z and y, the indication of the spiraling shape is performed and is computed as follows:

(27) $$z=r\times \cos\left(\theta \right)$$

(28) $$y=r\times \sin\left(\theta \right)$$

(29) $$r=r_{1}\times U\times D_{1}$$

(30) $$\theta =-w\times D_{1}+\theta_{1}$$

(31) $$\theta_{1}=\frac{3\pi }{2}$$

$r_{1}$ denotes a constant number within the range of [1, 10]. $D_{1}$ denotes an integer parameter range of [1, 10] again in the experiment. A constant parameter is again denoted by w and is fixed with a value of 0.01 in our experiment.

***Technique 3:*** This phase could be called the expanded exploitation phase. Sometimes during the exploitation phase, the target cannot be determined by the Aquila, and so complete reinitialization of the process must be performed. Then, the position must be updated with the help of the following equation:

(32) $$Y_{i}(t+1)=\lambda \times \left[Y_{best}(t)-Y_{p}(t)\right]+\sigma \times \left(U_{B}-L_{B}\right)\times r+L_{B}$$

where the lower-bound domain is indicated by $L_{B}$, and the upper-bound domain is indicated by $U_{B}$. The two fixed numbers or constants can be considered as λ and σ here.

***Technique 4:*** This phase could be called the forwarded exploitation phase. When the Aquila targets the prey by approaching it, it utilizes the focused exploitation technique by means of updating the exact position as follows:

(33) $$Y_{i}(t+1)=Q_{F}\times Y_{best}(t)-G_{1}\times Y_{i}(t)\times r-G_{2}\times Levy\left(D\right)+r\times G_{1}$$

where the quality factor, denoted by $Q_{F}$, helps to stabilize the search process and is calculated as follows:

(34) $$Q_{F}=t^{\frac{2\times r-1}{{\left(1-T\right)}^{2}}}$$

Once the position is updated, and the search process is stabilized and balanced, the best features are obtained and it is fed to the machine learning classifiers. As far as the fitness function is concerned here, it depends on FSR and classification errors such as the AOA.

### 3.4. Classification Using Two Hybrid Models

#### 3.4.1. ARIMA–AdaBoost Hybrid Model

With the help of the AdaBoost regressor, the regression inaccuracies can be easily tackled by AdaBoost techniques. The ARIMA regression model is hybrid with AdaBoost model and the classification is carried out [52]. The training instances can be reweighed successfully with the help of AdaBoost regressor so that weak regressors can be produced which could have a low variance error and high bias error. To obtain the final prediction, the hybridization of every weak regressor is performed so that a model can be obtained, which would have a low bias error and low variance.

Assuming a sample set such as $V=\left\{\left(a_{i},\left.b_{i}\right|i=1,2,\ldots ,N\right)\right\}$ is present initially.

At the $t^{th}$ iteration, the samples have a specific weight distribution and is specified by $D_{t(i),}t=1,2,\ldots ,T$.

The weight distribution is initialized during the initial iteration, i.e., when t=1, so that D1(i)=1N.

The forecasting error is computed as $e_{t(i)}=f_{t}(a_{i}),\left.b_{i}\right|,e_{t(i)}\in \left[0,1\right]$.

The expected output of $f_{t}(a_{i})$ is initially computed based on the weight distribution.

The proportional error computation is performed as $\varepsilon_{t}=\sum_{i=1}^{N}D_{t(i)}e_{t(i)}$.

The connection weights are traced out and are expressed as wt=12log1βt.

The weighing schemes are adapted accordingly and are expressed as

$D_{t+1,(i)}=\left(D_{t,(i)}\times \beta_{t}^{-\varepsilon_{t}}\right)/W_{t+1}$ and $W_{t+1}=\sum_{i=1}^{N}D_{t+1,(i)}$.

After a particular number of T iterations, the end predictions can be obtained as $F(a)=\sum_{t=1}^{T}w_{t}f_{t}(a)$.

A famous time-series forecasting method is the ARIMA model. Here, the ARIMA model is projected as $\left(k,d,l\right)$, where the autoregressive term is indicated by k, the moving average term is indicated by l, and the degree of difference is denoted by d, which helps to assess the stability of data. Structural models that are relatively complicated can be easily tackled by the ARIMA model. The future value of a variable is a simple linear combination of its errors, combined with its historical values and is expressed as

(35) $$P_{t}=\phi_{0}+\phi_{1}Q_{t-1}+\phi_{2}Q_{t-2}+\ldots \ldots +\phi_{k}Q_{t-k}+\varepsilon_{t}-\theta_{1}\varepsilon_{t-2},\ldots -\theta_{l}\varepsilon_{t-l}$$

where the actual value is represented by $P_{t}$.

The random error at t is indicated by $\varepsilon_{t}$, and the coefficients are indicated by $\phi_{i}$ and $\phi_{j}$. The autoregressive integer is specified by k, and the moving average integer is specified by l. The ARIMA $\left(k,d,l\right)$ has the important constituents of the model such as autoregression, integrated model and average motion. The time-series models between AR$\left(k\right)$ and MA$\left(l\right)$ have their own set of specifications. A simple AR model of order k, projected as AR$\left(k\right)$, is expressed as a linear process projected as follows:

(36) $$Q_{t}=c+\sum_{i=1}^{K}\phi_{i}Q_{t-i}+\varepsilon_{t}$$

where the constant is denoted as c. The coefficient of $Q_{t-i}$ is represented as $\phi_{i}$, and the white noise is represented as $\varepsilon_{t}$. The relationship between $Q_{t}$ values is the present period and the Q values in the past k periods are expressed by the AR$\left(k\right)$ model. For an order l, the moving average model is specified by the notation MA$\left(l\right)$ and is represented as

(37) $$p_{t}=\mu +\sum_{i=0}^{l}\theta_{i}\varepsilon_{t-i}$$

where the expected value of $p_{t}$ is denoted by μ. The Gaussian white noise is represented by $\varepsilon_{t}$ and the weight prescribed to the time period is expressed by $\theta_{0}=1$. The integer values help to replace the ARIMA $\left(k,d,l\right)$ model with the aid of three arguments of $\left(k,d,l\right)$. With changes in k,d and l, the generation of countless models can be achieved. The time attribute of the present and past data is used mainly for prediction purposes. To predict the future values successfully, a lot of patterns can be changed gradually during the process. With the help of an averaging technique, the classification of time series can be performed using this exponential smoothing technique. The null hypothesis can be investigated well with the help of the time-series data to analyze its stationary property.

For regression and classification tasks, AdaBoost can be used, as it is a versatile framework. Based on a specific number of rules, many weak estimators are used in this framework. During the training process of the AdaBoost classifier, decision trees and regression trees are used as a single learning technique. The important steps for the ARIMA–AdaBoost classification are as follows:

The collection of data and assimilation, performed together so that the train and test sets can be decomposed easily;

The implementation of the stationarity test;

Regression model training using AdaBoost;

The validation of the generated model using a test dataset.

**Figure 2:**
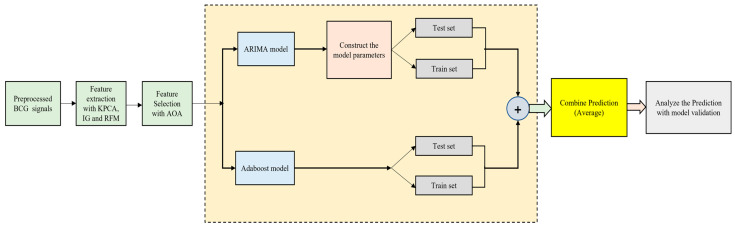
Simplified illustration of the ARIMA–AdaBoost Hybrid model.

This model can be applied to other datasets and real-world issues as well. The simplified flowchart of the classification model of the ARIMA–AdaBoost hybrid classifier is shown in Figure 2 as follows:

#### 3.4.2. Time-Weighted Hybrid AdaBoost–Support Vector Machine (TW-HASVM) Model

Based on time, the data batches are given more importance, and so this approach is termed the time-weighting approach [53]. The formulation of time-weighing function is expressed as follows:

(38) $$TW_{t}=\exp\left(-\eta t\right),t=0,1,2,\ldots ,H-1$$

The batch number of the data is represented as t, and the highest number of data batch is represented as H, respectively. The value of η is defined from 0 to 0.5 in this work. The sample weighting model is improved by the TW-HASVM classification model. The sample weighting functions are greatly improvised and hybridized by using the average sample weights and are represented as follows:

(39) $$TW_{1}^{k}=\frac{1}{n},k=1,2,\ldots ,N$$

(40) $$TW_{u+1}^{k}=TW_{u}^{k}\exp\left(-\gamma_{u}l_{u}^{k}\exp\left(\gamma t^{k}l_{u}^{k}\right)\right),\left(k=1,2,\ldots ,n;u=1,2,\ldots ,u-1\right)$$

(41) $$l_{u}^{k}=\left\{\begin{array}{lll} 1 & if & b^{k}=a^{k}\\ -1 & if & b^{k}\neq a^{k} \end{array}\right.$$

The highest number of iterations are indicated by u, and the total number of samples is represented by n. The weight factor is represented by $\gamma_{u}$. $TW_{u+1}^{k}$ represents the weight of the $u+1^{th}$ iteration. The term $l_{u}^{k}$ represents the control parameter of the sample weight direction. For the misclassified samples, the value is expressed as −1, and for correctly classified samples, the value is expressed as +1. For the classifier, the voting weight is expressed as follows:

(42) $$\gamma_{u}=0.5\ln\left[\frac{1-\varepsilon_{u}}{\varepsilon_{u}}\right]$$

This time weighted concept is incorporated with the SVM–AdaBoost hybrid classifier as follows. SVM always tries to trace the optimal hyperplane so that the data points can be separated well in various classes [54]. With the aid of kernel functions, the data is transferred to high dimensional spaces by the SVM. In AdaBoost, the weak classifiers are trained iteratively, and the data points are constantly adjusted so that the performance of the weak classifier is improved. Higher weights are assigned to the misclassified instances so that the preceding weak classifiers have a higher focus on other weak training samples. By hybridization of these two techniques, the design of the TW-HASVM classifier is performed so that the data imbalance problem can be solved well. In this hybrid model, multiple SVM classifiers are trained by the AdaBoost algorithm on various subsets. To the specific data points, the time weights are introduced so that the higher weights are received by the hybrid form. The distribution of the data becomes relatively sensitive in the classification process, and the assignment of time weights largely stabilizes the process. The hybrid weighted model of the SVM classifiers, when trained with the AdaBoost algorithm, yields the hybrid TW-HASVM classifier. Handling the imbalance data becomes much easier with his hybrid classifier rather than the traditional one, as a versatile performance could be maintained in the majority class.

The training of the TW-HASVM hybrid classifier is performed with the help of the chosen features. Multiple SVM classifiers are trained with the help of AdaBoost algorithm on various subsets of the data. The assignment of the various time weights is performed on the weight points. Weights which are higher are received by the recent instances, and weights which are lower are received by the later instances. In the data distribution, a lot of recent changes can be captured by this hybrid classifier. In the training dataset, for each data point, the time weights $w_{i}^{\left(1\right)}$ are initialized. The weight assignment process is carried out successfully and is represented as follows:

(43) wi(1)=1n for i=1,2,…,n

In the training dataset, every data point i is prioritized with an initial weight of 1n, where the data point number is indicated by n. For the AdaBoost algorithm, the number of iterations must be determined. This helps to assess the training of weak SVM classifiers. For all the iterations $\left(t=1,2,\ldots ,T\right)$, the time weights are normalized as follows:

$w_{i}^{\left(t\right)}=w_{i}^{\left(t\right)}/N_{t}$, where the normalization factor is represented as $N_{t}$ and is indicated as follows:

(44) $$N_{t}=sum\left(w_{i}^{\left(t\right)}\;for\;i=1,2,\ldots ,n\right)$$

Depending on the normalized time weights, the training data subset is sampled so that the weak SVM classifiers can be trained well in every iteration. With the help of the kernel function, the SVM classifier is trained $\left(C_{t}\right)$, and in this work, a polynomial kernel is used. On the entire training dataset, the performance of the weak classifier is assessed, and the classification error rate is computed. When computing the classification error rate, the time weights are taken into consideration and are represented as follows:

(45) $$e_{t}=sum\left(w_{i}(t)*I\left(b_{i}\neq C_{t}\left(a_{i}\right)\right)\right)/sum\left(w_{i}(t)\right)$$

where the indicator function is represented by I, and a misclassification is represented by $b_{i}\neq C_{t}(a_{i})$. The classifier weight $\alpha_{t}$ is calculated as a projection of error rate $\left(e_{t}\right)$. The computation of weight is as follows:

(46) $$\alpha_{t}=0.5*\log\left(\left(1-e_{t}\right)/e_{t}\right)$$

To the classifiers, the assignment of the classifier weight is performed so that a low error rate is obtained. The updating of time weights of instances is performed in the AdaBoost algorithm at every iteration so that the performance of the weak classifiers is upgraded well. The updating of time weights is expressed as follows:

(47) $$w_{i}(t+1)=w_{i}(t)*\exp\left(\alpha_{t}*I\left(b_{i}\neq C_{t}(a_{t})\right)\right)\;for\begin{array}{c} \;\;\;\;\;i=1,2,\ldots ,n \end{array}$$

The data point i has a particular time weight at a specific iteration t and is represented as $w_{i}(t)$. The data point i is now updated with a new time weight and ready for the upcoming iteration $\left(t+1\right)$, being represented as $w_{i}(t+1)$. For the upcoming weak classifiers, the training data is sampled by the updated weight. At a specific iteration t, based on a weak classifier $\left(C_{t}\right)$, a particular weight is assigned to improve its performance and is expressed by $\alpha_{t}$. $I\left(b_{i}\neq C_{t}(a_{t})\right)$ represents the indicator function to analyze the difference between time class label $b_{i}$ and the predicted class label $C_{t}\left(a_{t}\right)$. The indicator function expresses the output as 0 if the prediction is correct, and the indicator function expresses the output as 1 if the prediction is incorrect. The following expression is utilized to compute the product of the indicator function I and the weight of the classifier $\alpha_{t}$.

(48) $$\exp\left(\alpha_{t}*I\left(b_{i}\neq C_{t}\left(a_{t}\right)\right)\right)$$

There will be a large exponential term if there is an incorrect prediction, so that in the next iteration the time weight will be increased. The exponential term equals 1 if there is a correct prediction, so that in the next iteration the time weight remains steady.

Hybridization classifiers.

Due to the weighted amalgamation of the independent SVM classifiers trained with the aid of the AdaBoost algorithm, the TW-HASVM classifier is obtained. During the training process, the performance measure obtained is assessed, and then the determination of every weight for the SVM classifier is performed. The higher weights received by the best classifier are assessed well in this model. As a weighted hybridization of all the weak SVM classifiers along with their weights, the ultimate TW-HASVM classifier is obtained after finishing all the iterations and is represented as follows:

(49) $$F(a)=sum\left(\alpha_{t}*C_{t}(a)\right)\;for\;t=1,2,\ldots ,T$$

Classification process by TW-HASVM:

Once the training of the TW-HASVM is performed, it is utilized to assess and classify the features. The output of every SVM classifier is computed when the features are presented to it, and it is performed in the weighted combination. With the help of a weighted vote, the assessment of a final class label is performed. The final prediction is nothing but the class which has obtained the largest combined weight in the classification model. Assuming the data points as a, the TW-HASVM hybrid classifier aims to classify and predict its class label. The output for the data point $\left(a\right)$ is computed for all the weak SVM classifiers $\left(C_{t}\right)$. The prediction of the class label by the weak classifier $\left(C_{t}\right)$ is the respective output and is represented as follows:

(50) $$h_{t}(a)=C_{t}(a)$$

For every class label, the weighted output sum is calculated with the help of classifier weights $\left(\alpha_{t}\right)$ and is represented as follows:

(51) $$S_{c_{l}}(a)=sum\left(\alpha_{t}*h_{t}(a)\right)\;for\;c_{l}\in \left\{1,2,\ldots ,c_{l}\right\}$$

where the number of classes is expressed by $c_{l}$. Depending on the number of classes, the weighted sum for every class can be computed. For the data point $\left(a\right)$, the final class label is analyzed by means of choosing the class which has the highest weighted sum. The hybrid classifier’s prediction is this class label.

(52) $$b_{pred}(a)=\arg\max_{c_{l}}\left(S_{c_{l}}\left(a\right)\right)$$

where each class is represented by the label $c_{l}$. The weighted output sum is indicated by $S_{c_{l}}(a)$ and is computed as follows:

(53) $$S_{c_{l}}(a)=sum\left(\alpha_{t}*h_{t}(a)\right)\;for\;c_{l}\in \left\{1,2,\ldots ,c_{l}\right\}$$

**Figure 3:**
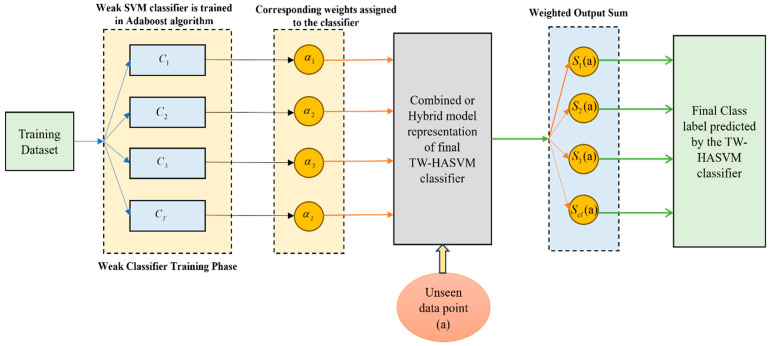
Simplified illustration of the TW-HASVM classification model.

The number of classes is represented by $c_{l}$. The term $h_{t}(a)$ represents the output of the weak SVM classifier $C_{t}$. At an iteration t and for the data point a, the classifier weight is expressed as $\alpha_{t}$. The overall simplified illustration of the TW-HASVM classification model is expressed in Figure 3 as follows.

## 4. Results and Discussion

A publicly available BCG database was used for the analysis in this work [29]. The explanation of the dataset is provided in detail as follows. For the acquisition of BCG signals, a standard BCG signal acquisition termed RS-611 was used in this study [29,55]. The RS-611 comprises three important components, such as a sensitive mattress which can track the micro-movements, an analog-to-digital converter (ADC), and a personal computer attached to it. There are two main hydraulic pressure sensors attached to the sensitive mattress, where the upper part of the sensor is attached to the chest and the lower part of sensor is attached to the leg. Both components are placed in a parallel manner so that the BCG signal can be captured easily from the subjects. The original pressure caused due to the various cardiac activities is recorded, amplified and then, with the help of the ADC device, it is converted to a 16-bit resolution digital signal which had a sample rate of 100 Hz. The main advantage of using RS-611 in the experimentation study [29,55] is because it does not have any complexity in terms of handling and maintenance.

A total of 175 people were initially considered as subjects in this study. Before recruiting them for this study, their informed consent was obtained for this study, and it was performed according to the standard medical experimental norms of the Helsinki Declaration standards. To obtain the basic physiological index, preliminary routine examinations were conducted. With the help of a mercury sphygmomanometer, the measurement of blood pressure was performed in the left arm consecutively with short intervals. Based on the standard set by the American Society of Hypertension referred to in [56], the measurement was performed when the subjects were seated in a relaxed position. The recording of the blood pressure was performed in different sessions in a time span of three weeks, and then the values obtained were arranged so that the final systolic and diastolic values of the blood pressure were obtained. If the subjects had a blood pressure reading greater than 140/90 mm Hg, it was categorized as hypertensive; otherwise, it was categorized as normal. The subjects with the following criteria were excluded from the experimentation procedure: subjects using anti-hypertensive medication when the blood pressure was measured, subjects with a clinical history of myocardial infarction, subjects with a Body Mass Index (BMI) > 30, subjects with diabetes mellitus, subjects with smoking and chronic alcoholism and subjects with atrial fibrillation conditions. Once these exclusions were incorporated, a total of 68 hypertensive classes and 76 normal classes were shortlisted. Again, after further scrutiny, some subjects were eliminated. One day before the experiment was conducted, the subjects were asked to sleep well by means of adjusting the room temperature, light intensity and noise levels so that they could sleep peacefully for a period of about 7 to 8 h. The next morning, these subjects were asked some questions about their sleep pattern. The BCG recordings of the subjects who reported that they did not sleep well or had a disturbed sleep the night before the experimentation day were deemed unfit for research and were discarded. A manual investigation was conducted into the BCG recordings, and some recordings, which showed drastic fluctuations, were eliminated. The final BCG dataset was obtained from a total of 128 participants, among whom there were 67 normal subjects and 61 subjects with hypertension. At a frequency of 100 Hz, the BCG signals were sampled in this dataset. The initial segmentation of the BCG signals was performed for a period of 5 min, and in this work the basic preprocessing step of utilizing ICA to the BCG signals was implemented before proceeding with the proposed pragmatic models. The dataset can be accessed at the following URL: https://figshare.com/articles/dataset/Unobtrusive_Mattressbased_Identification_of_Hypertension_by_Integrating_Classification_and_Association_Rule_Mining/7594433. The details of the dataset are provided in Table 1 and Table 2 below.

Using the MATLAB R2022a platform, the experiment was conducted on a system which had a 64 bit operating system, 16 GB of RAM and a 1.60 GHz processor. A 10-fold cross-validation scheme was utilized thoroughly for all the hybrid classifiers used in this work. For the KNN algorithm used in this work, the value of k was chosen to be 10 in our experiment. For the KPCA algorithm used in this work, the kernel type was set to linear, and the total number of components used was four. For the TW-HASVM classifier, the current data batch number was set to 1, and the weights were adjusted accordingly. For the BTSA, the hyperparameter settings were set as follows: the search agents were set to 100, and the number of generations was set to 1000. The minimum parameters and maximum parameters were assigned in the range of 1 to 20 for this algorithm. The exploitation fixed parameters in AOA were set to 0.5, respectively. The total number of iterations used in the AdaBoost module was set to 1000, and the kernel type used when engaging the SVM classifier was a polynomial kernel. For comparison purposes, other prominent feature selection techniques such as the chi-square test, Pearson’s correlation coefficient (PCC), Particle Swarm Optimization (PSO) and Ant Colony Optimization (ACO) were considered. In addition, for the comparison of machine learning performance, the standard machine learning classifiers such as RF, Decision Trees (DTs), Naïve Bayesian Classifier (NBC) and the standard AdaBoost were considered apart from the concentrated four hybrid classifiers in this work. The main performance metric analyzed was classification accuracy and was represented in terms of True Positive (TP), True Negative $\left(TN\right)$, False Positive $\left(FP\right)$, False Negative (FN) and computed as follows:

(54) $$Accuracy=\frac{TP+TN}{TP+TN+FP+FN}$$

On examining Table 3, a high classification accuracy of 96.48% is observed when the MODWT feature extraction technique is combined with the BTSA feature selection technique and classified with the Hybrid AdaBoost–MULDA classifier. On careful analysis of Table 4, a high classification accuracy of 93.87% is observed when the MODWT feature extraction technique is combined with the IG feature selection technique and classified with the Hybrid AdaBoost–MULDA classifier. When Table 5 is examined, a high classification accuracy of 87.36% is observed when the MODWT feature extraction technique is combined with the chi-squared feature selection technique and classified with the Hybrid AdaBoost–MULDA classifier. If Table 6 is studied, a high classification accuracy of 89.9% is observed when the EWT feature extraction technique is combined with the PCC feature selection technique and classified with the Hybrid AdaBoost–MULDA classifier. If Table 7 is carefully studied, then, a high classification accuracy of 91.23% is observed when the MODWT feature extraction technique is combined with the PSO feature selection technique and classified with the Hybrid AdaBoost–MULDA classifier. On examining Table 8, a high classification accuracy of 89.67% is observed when the MODWT feature extraction technique is combined with the ACO feature selection technique and classified with the Hybrid AdaBoost–MULDA classifier. On careful analysis of Table 9, a high classification accuracy of 96.89% is observed when the KPCA feature extraction technique is combined with the AOA feature selection technique and classified with the Hybrid ARIMA–AdaBoost classifier. When Table 10 is examined, a high classification accuracy of 92.37% is observed when the KPCA feature extraction technique is combined with the IG feature selection technique and classified with the TW-HASVM classifier. On examining Table 11, a high classification accuracy of 89.30% is observed when the RFM feature extraction technique is combined with the chi-square feature selection technique and classified with the TW-HASVM classifier. If Table 12 is studied, a high classification accuracy of 88.88% is observed when the KPCA feature extraction technique is combined with the PCC feature selection technique and classified with the TW-HASVM classifier. On examining Table 13, a high classification accuracy of 91.48% is observed when the RFM feature extraction technique is combined with the PSO feature selection technique and classified with the Hybrid ARIMA–AdaBoost classifier. On careful analysis of Table 14, a high classification accuracy of 89.84% is observed when RFM feature extraction technique is combined with the ACO feature selection technique and classified with the Hybrid ARIMA–AdaBoost classifier.

**Figure 4:**
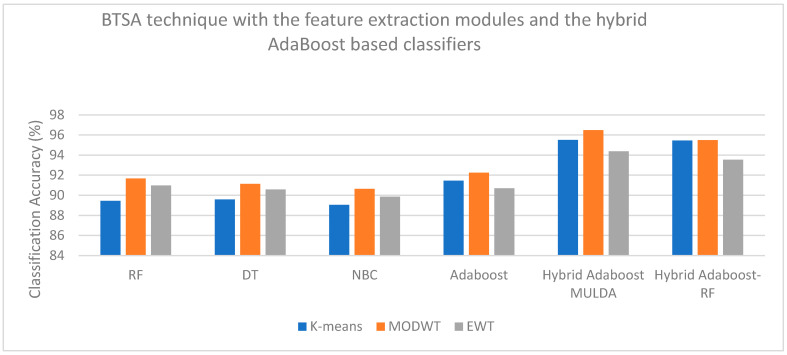
Comparison of performance analysis (accuracy) of the BTSA feature selection technique combined with the feature extraction modules and the hybrid AdaBoost-based classifiers.

**Figure 5:**
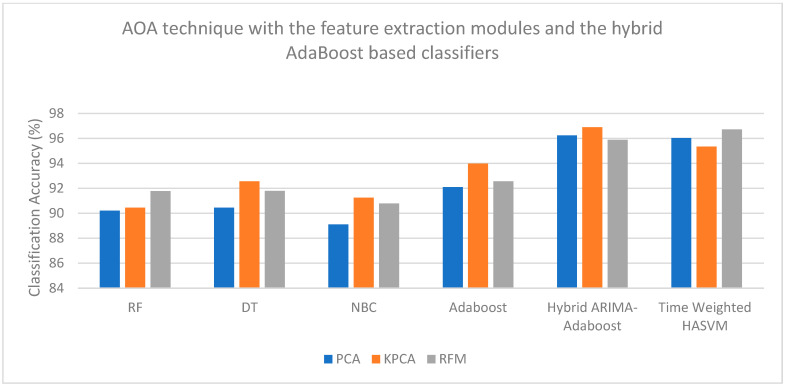
Comparison of performance analysis (accuracy) of the AOA feature selection technique combined with the feature extraction modules and the hybrid AdaBoost-based classifiers.

**Figure 6:**
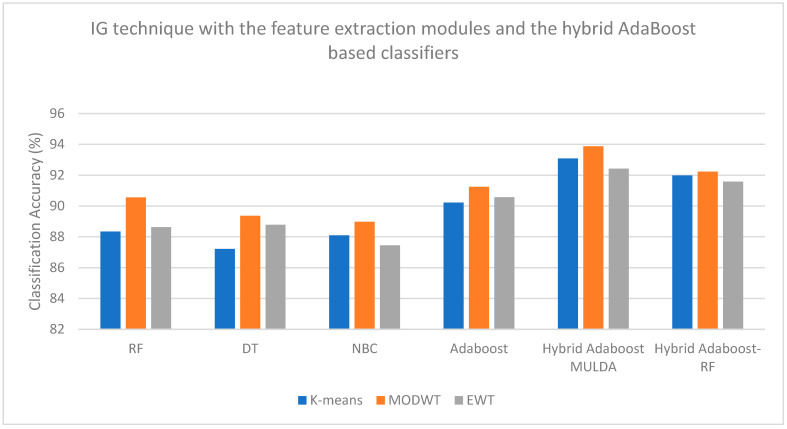
Comparison of performance analysis (accuracy) of the IG feature selection technique combined with the feature extraction modules and the hybrid AdaBoost-based classifiers.

**Figure 7:**
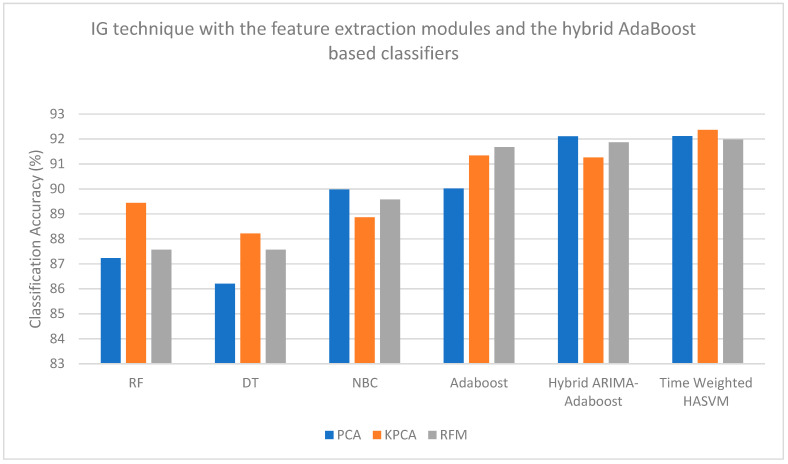
Comparison of performance analysis (accuracy) of the IG feature selection technique combined with the feature extraction modules and the hybrid AdaBoost-based classifiers.

Figure 4, Figure 5, Figure 6 and Figure 7 are plotted here, as they yield comparatively higher classification accuracies than the remaining methods. The overall higher classification accuracy is yielded by the AOA feature selection technique and the Hybrid ARIMA–AdaBoost classification model. The second highest classification accuracy is achieved by the BTSA feature selection technique and the Hybrid AdaBoost–MULDA classification model. The third highest classification accuracy is achieved by the IG feature selection technique employed with the MODWT feature extraction model and classified with the Hybrid AdaBoost–MULDA classification model. The fourth highest classification accuracy is achieved by the IG feature selection technique combined with the MODWT feature extraction scheme and the TW-HASVM classification model.

When the standard Cohen’s Kappa coefficient test was applied to the extracted features, the values indicated good agreement, ranging from 0.5 to 1, respectively. A good variation was found for the feature values when Friedman test analysis was conducted, and so it was deemed fit for the classification. The extracted and selected features passed the standard Kruskal–Wallis test, with statistically significant results (p<0.01), indicating that the features were correctly extracted and retained the original information; therefore, the features can be passed through the hybrid classifiers. When a two-sided Wilcoxon test was applied, all features showed statistically significant effects. As far as computational complexity was concerned, all the models exhibited low complexity and were therefore analyzed as $O\left(n^{3}\log n\right)$.

### 4.1. Comparison with Previous Works

For a clearer comparison, the results of the current study are compared with previous work and tabulated in Table 15.

In this work, an exhaustive analysis of BCG signals was conducted for the classification of hypertension using multiple feature extraction techniques, multiple feature selection schemes and multiple machine learning classifiers, including a few hybrid machine learning models. The results obtained yielded comparatively good results when compared to a majority of previous studies. Though some previous works have obtained a slightly higher classification accuracy than the present work, it must be noted that the current work has proposed plenty of methods, and a comprehensive analysis was conducted, which many previous studies failed to perform, focusing only on a single proposed method. Considering this aspect and the workflow of the paper, this work is considered good in terms of an exhaustive analysis performed on the BCG signals for the classification of hypertension.

The overall computational time for the best performing models was computed and is shown in Table 16 below:

On analysis of Table 16, it is evident that a low computational time of 5.112 s was produced by the model which employs the KPCA feature extraction technique, the AOA feature selection scheme and the Hybrid ARIMA–AdaBoost classifier. The next best computational time of 6.012 s was achieved by the model which employs the RFM feature extraction technique, the AOA feature selection scheme and the Hybrid ARMIA–AdaBoost classifier.

### 4.2. Limitations of This Work

For a research framework like this, no exact physiological rationale can be provided as to why only some feature extraction/selection techniques along with certain machine learning classifiers were chosen in the experiment. With hundreds of well-known feature extraction methods and thousands of feature selection techniques available in the literature, it is very difficult to justify why only a particular subset of methods was considered for this research. During the analysis, when starting to build the experiment, various factors were considered in the research, as follows: the combination should not have been employed by other researchers in the past, the workflow should be novel to some extent and it should be the first of its kind to have been applied to the BCG dataset so that the results can be analyzed well. Based on these factors, the experimentation was performed by choosing some random algorithms and evaluating their performance on the BCG dataset.

The two pragmatic models are not two distinct models but rather two large baskets of arbitrarily grouped techniques. In this research, the flow is presented as a mechanical pipeline, and the major insight here is very simple: just an ordinary flow of feature extraction and feature selection, followed by classification. The results can be attributed to some factors, as they lie in the effectiveness of the algorithms used and it is always interesting to observe how a particular set of feature extraction techniques, when selected with conventional feature selection techniques and metaheuristic optimizers, followed by classification with traditional and hybrid classifiers, perform for a particular dataset to yield a specific set of results. The reason for the classification accuracy may be attributed to the intrinsic properties of the wonderful algorithms used, as these algorithms work independently and depend on the hyperparameters set by the authors. Based on the natural design of the algorithm and the hyperparameters set by the authors, the results can be obtained.

Moreover, another limitation is that the discrimination improvement obtained by adding BCG-derived features to a model including all clinical characteristics alone, compared with the clinical model by itself, was not thoroughly investigated in this work, and this will be considered as possible future work. The addition of BCG-derived parameters to patient-factor–based models improved the prediction of hypertension, but the incremental predictive performance, with discrimination analysis and the benefit of BCG parameters for identifying individuals with hypertension, was not analyzed in detail in this work, and it could be considered as possible future work. In this work, only classification accuracy is given a high priority, and in the future, more biomedical metrics are planned to be computed, such as sensitivity, specificity, precision, F1-score, geometric mean, etc.

## 5. Conclusions and Future Works

In the tele-healthcare paradigm, an important achievement has been made in the field of Internet of Medical Things and medical signal processing. When the blood circulates in the body, a ballistic force is generated, and it can be assessed as a BCG signal. Various sensors were utilized in the past for extracting the BCG signal, as it is more flexible and provides greater convenience to use. In this work, two interesting pragmatic models using efficient feature extraction, feature selection and hybrid machine learning models are used for the analysis and classification of BCG signals for the assessment of hypertension. The best results are shown when the KPCA feature extraction technique is used with the AOA feature selection technique and classified with the Hybrid ARIMA–AdaBoost classifier, reporting a classification accuracy of 96.89%. The second-best classification accuracy of 96.48% is obtained when the MODWT feature extraction technique is used with the BTSA feature selection technique and classified with the Hybrid AdaBoost–MULDA classifier. The third-best classifier classification accuracy of 96.23% is obtained when the PCA feature extraction technique is used with the AOA feature selection technique and classified with the Hybrid ARIMA–AdaBoost classifier. Future work plans to use a variety of other efficient feature extraction techniques, other novel metaheuristic and swarm-based algorithms for feature selection and more efficient hybridization of machine learning classifiers so that the intrinsic properties of all the classifiers can be utilized well, and hence the classification accuracy can be improved to a much higher extent. Future work also plans to incorporate the concept of explainable Artificial Intelligence (EAI), along with the pragmatic models to test its efficacy in terms of classification accuracy. In addition, the work is planned to be implemented on real-time test beds, and it can be applied to telemedicine applications as well in future work.

## Figures and Tables

**Table 1:** Hypertensive group.

**Number**	61
**Gender (male)**	33
**Gender (female)**	38
**Age group**	55.6 ± 7.9
**Body Mass Index (BMI)**	24.3 ± 3.6
**Heart rate (bpm)**	77.1 ± 9.2
**Systolic blood pressure (mmHg)**	155.6 ± 11.2
**Diastolic blood pressure (mmHg)**	103.6 ± 8.2

**Table 2:** Control group.

**Number**	67
**Gender (male)**	35
**Gender (female)**	32
**Age group**	53.2 ± 9.2
**Body Mass Index (BMI)**	23.4 ± 3.3
**Heart rate (bpm)**	73.6 ± 8.3
**Systolic blood pressure (mmHg)**	112.1 ± 15.7
**Diastolic blood pressure (mmHg)**	74.4 ± 6.3

**Table 3:** Performance analysis (accuracy) of the BTSA feature selection technique combined with the feature extraction modules and the hybrid AdaBoost-based classifiers.

	RF	DT	NBC	AdaBoost	Hybrid AdaBoost–MULDA	Hybrid AdaBoost–RF
**K-means**	89.45	89.58	89.05	91.45	95.51	95.45
**MODWT**	91.67	91.14	90.64	92.24	96.48	95.48
**EWT**	90.98	90.57	89.87	90.69	94.37	93.54

**Table 4:** Performance analysis (accuracy) of the IG feature selection technique combined with the feature extraction modules and the hybrid AdaBoost-based classifiers.

	RF	DT	NBC	AdaBoost	Hybrid AdaBoost–MULDA	Hybrid AdaBoost–RF
**K-means**	88.34	87.21	88.09	90.22	93.09	91.98
**MODWT**	90.56	89.36	88.97	91.24	93.87	92.23
**EWT**	88.63	88.78	87.45	90.57	92.42	91.58

**Table 5:** Performance analysis (accuracy) of the chi-square feature selection technique combined with the feature extraction modules and the hybrid AdaBoost-based classifiers.

	RF	DT	NBC	AdaBoost	Hybrid AdaBoost–MULDA	Hybrid AdaBoost–RF
**K-means**	85.09	82.21	83.87	84.33	86.21	85.02
**MODWT**	84.97	83.24	84.74	81.37	87.36	86.34
**EWT**	83.58	84.67	85.35	83.89	86.98	85.67

**Table 6:** Performance analysis (accuracy) of the PCC feature selection technique combined with the feature extraction modules and the hybrid AdaBoost-based classifiers.

	RF	DT	NBC	AdaBoost	Hybrid AdaBoost–MULDA	Hybrid AdaBoost–RF
**K-means**	84.23	80.09	84.21	86.09	88.11	88.05
**MODWT**	83.45	81.87	82.25	83.98	89.26	89.04
**EWT**	83.68	82.34	84.78	85.42	89.90	87.37

**Table 7:** Performance analysis (accuracy) of the PSO feature selection technique combined with the feature extraction modules and the hybrid AdaBoost-based classifiers.

	RF	DT	NBC	AdaBoost	Hybrid AdaBoost–MULDA	Hybrid AdaBoost–RF
**K-means**	86.32	84.89	82.21	84.09	90.21	89.33
**MODWT**	87.66	85.97	83.25	86.98	91.23	90.46
**EWT**	88.78	85.55	82.78	85.77	90.58	88.78

**Table 8:** Performance analysis (accuracy) of the ACO feature selection technique combined with the feature extraction modules and the hybrid AdaBoost-based classifiers.

	RF	DT	NBC	AdaBoost	Hybrid AdaBoost–MULDA	Hybrid AdaBoost–RF
**K-means**	85.21	84.03	85.21	87.03	89.21	87.01
**MODWT**	85.33	86.45	83.23	84.33	89.67	88.21
**EWT**	85.48	86.68	82.69	85.59	87.99	87.08

**Table 9:** Performance analysis (accuracy) of the AOA feature selection technique combined with the feature extraction modules and the hybrid AdaBoost-based classifiers.

	RF	DT	NBC	AdaBoost	Hybrid ARIMA–AdaBoost	TW-HASVM
**PCA**	90.21	90.44	89.11	92.09	96.23	96.02
**KPCA**	90.45	92.56	91.24	93.98	96.89	95.34
**RFM**	91.78	91.79	90.78	92.56	95.89	96.71

**Table 10:** Performance analysis (accuracy) of the IG feature selection technique combined with the feature extraction modules and the hybrid AdaBoost-based classifiers.

	RF	DT	NBC	AdaBoost	Hybrid ARIMA–AdaBoost	TW-HASVM
**PCA**	87.23	86.21	89.98	90.02	92.11	92.12
**KPCA**	89.44	88.22	88.87	91.34	91.26	92.37
**RFM**	87.57	87.57	89.58	91.68	91.87	91.98

**Table 11:** Performance analysis (accuracy) of the chi-square feature selection technique combined with the feature extraction modules and the hybrid AdaBoost-based classifiers.

	RF	DT	NBC	AdaBoost	Hybrid ARIMA–AdaBoost	TW-HASVM
**PCA**	84.21	80.78	84.09	85.21	87.22	84.22
**KPCA**	83.22	81.98	85.87	82.35	88.37	85.37
**RFM**	81.46	82.36	83.36	84.78	86.65	89.30

**Table 12:** Performance analysis (accuracy) of the PCC feature selection technique combined with the feature extraction modules and the hybrid AdaBoost-based classifiers.

	RF	DT	NBC	AdaBoost	Hybrid ARIMA–AdaBoost	TW-HASVM
**PCA**	82.89	82.12	82.09	84.21	86.87	87.09
**KPCA**	80.44	80.34	81.84	85.25	85.77	88.88
**RFM**	80.57	81.78	83.22	84.67	87.63	87.34

**Table 13:** Performance analysis (accuracy) of the PSO feature selection technique combined with the feature extraction modules and the hybrid AdaBoost-based classifiers.

	RF	DT	NBC	AdaBoost	Hybrid ARIMA–AdaBoost	TW-HASVM
**PCA**	87.21	86.87	85.09	88.23	90.77	89.21
**KPCA**	88.22	87.75	86.87	85.45	90.63	91.36
**RFM**	89.36	87.49	84.34	87.61	91.48	89.89

**Table 14:** Performance analysis (accuracy) of the ACO feature selection technique combined with the feature extraction modules and the hybrid AdaBoost-based classifiers.

	RF	DT	NBC	AdaBoost	Hybrid ARIMA–AdaBoost	TW-HASVM
**PCA**	83.21	85.09	82.23	85.09	88.24	89.09
**KPCA**	82.34	84.36	84.67	86.97	88.67	87.88
**RFM**	84.98	83.78	85.33	84.45	89.84	89.21

**Table 15:** Performance summary of the current results compared with previous studies.

Year	Authors	Methods	Performance Metrics
2018	Liu et al. [28]	Mining class association from multi-dimensional features	Acc = 85.20%
2019	Liu et al. [29]	Integrated classification and association rule mining	Acc = 84.40%
2021	Rajput et al. [31]	EMD, wavelet decomposition and nonlinear techniques	Acc = 89.00%
2022	Rajput et al. [32]	Continuous wavelet transform combined with deep neural networks	Acc = 86.14%
2022	Gupta et al. [33]	Gabor transform and deep convolutional neural network	Acc = 97.65%
2023	Gupta et al. [39]	Integrated tunable Q-factor wavelet transform and multiverse optimization concept	Acc = 92.21%
2023	Ozcelik et al. [38]	Spectrogram tasks + ConvMixer	Acc = 97.69%
2024	Dogan et al. [37]	Odd–even pattern with KNN algorithm and majority voting	Acc = 97.78%
2025	***Proposed works***	(a) K-means + BTSA and Hybrid AdaBoost–MULDA (b) MODWT + BTSA + Hybrid AdaBoost–MULDA (c) EWT + BTSA + Hybrid AdaBoost–MULDA	Acc = 95.51% Acc = 96.48% Acc = 94.37%
		(a) K-means + IG and Hybrid AdaBoost–MULDA (b) MODWT + IG + Hybrid AdaBoost–MULDA (c) EWT + IG + Hybrid AdaBoost–MULDA	Acc = 93.09% Acc = 93.87% Acc = 92.42%
		(a) PCA + AOA and Hybrid ARIMA–AdaBoost (b) KPCA + AOA + Hybrid ARIMA–AdaBoost (c) RFM + AOA + Hybrid ARIMA–AdaBoost	Acc = 96.23% Acc = 96.89% Acc = 95.89%
		(a) PCA + IG + TW-HASVM (b) KPCA + IG + TW-HASVM (c) RFM + IG + TW-HASVM	Acc = 92.12% Acc = 92.37% Acc = 91.98%

**Table 16:** Computational time analysis for the best performing models.

Year	Authors	Methods	Computational Time
2025	***Proposed works***	(d) K-means + BTSA and Hybrid AdaBoost–MULDA (e) MODWT + BTSA + Hybrid AdaBoost–MULDA (f) EWT + BTSA + Hybrid AdaBoost–MULDA	10.563 s 11.342 s 11.291 s
		(d) K-means + IG and Hybrid AdaBoost–MULDA (e) MODWT + IG + Hybrid AdaBoost–MULDA (f) EWT + IG + Hybrid AdaBoost–MULDA	10.247 s 9.298 s 9.037 s
		(d) PCA + AOA and Hybrid ARIMA–AdaBoost (e) KPCA + AOA + Hybrid ARIMA–AdaBoost (f) RFM + AOA + Hybrid ARIMA–AdaBoost	6.567 s 5.112 s 6.012 s
		(d) PCA + IG + TW-HASVM (e) KPCA + IG + TW-HASVM (f) RFM + IG + TW-HASVM	8.378 s 9.001 s 8.981 s

## Data Availability

The dataset can be accessed at the following URL: https://figshare.com/articles/dataset/Unobtrusive_Mattress-based_Identification_of_Hypertension_by_Integrating_Classification_and_Association_Rule_Mining/7594433.
